# A Naturally Associated Rhizobacterium of Arabidopsis thaliana Induces a Starvation-Like Transcriptional Response while Promoting Growth

**DOI:** 10.1371/journal.pone.0029382

**Published:** 2011-12-28

**Authors:** Jens Schwachtje, Silke Karojet, Ina Thormählen, Carolin Bernholz, Sabine Kunz, Stephan Brouwer, Melanie Schwochow, Karin Köhl, Joost T. van Dongen

**Affiliations:** Max Planck Institute of Molecular Plant Physiology, Potsdam-Golm, Germany; University of Helsinki, Finland

## Abstract

Plant growth promotion by rhizobacteria is a known phenomenon but the underlying mechanisms are poorly understood. We searched for plant growth-promoting rhizobacteria that are naturally associated with *Arabidopsis thaliana* to investigate the molecular mechanisms that are involved in plant growth-promotion. We isolated a *Pseudomonas* bacterium (*Pseudomonas sp.* G62) from roots of field-grown *Arabidopsis* plants that has not been described previously and analyzed its effect on plant growth, gene expression and the level of sugars and amino acids in the host plant. Inoculation with *Pseudomonas sp.* G62 promoted plant growth under various growth conditions. Microarray analysis revealed rapid changes in transcript levels of genes annotated to energy-, sugar- and cell wall metabolism in plants 6 h after root inoculation with *P. sp.* G62. The expression of several of these genes remained stable over weeks, but appeared differentially regulated in roots and shoots. The global gene expression profile observed after inoculation with *P. sp.* G62 showed a striking resemblance with previously described carbohydrate starvation experiments, although plants were not depleted from soluble sugars, and even showed a slight increase of the sucrose level in roots 5 weeks after inoculation. We suggest that the starvation-like transcriptional phenotype - while steady state sucrose levels are not reduced - is induced by a yet unknown signal from the bacterium that simulates sugar starvation. We discuss the potential effects of the sugar starvation signal on plant growth promotion.

## Introduction

Sessile plants are generally associated with soil microorganisms. Interactions between plants and fungi or bacteria can be mutualistic and therefore beneficial for plant fitness. Mycorrhizal interactions with plants have been studied in great detail and extensive knowledge exists about the beneficial physiological and molecular interaction, and communication [1], [2], [3]. Likewise, interactions between plants and nitrogen-fixing bacteria are very well investigated. Best understood is the plant-bacterial interaction between legumes and rhizobia [4], [5], [6], [7], while increasing knowledge is being obtained on the association between nitrogen fixing bacteria and various graminaceous species [8], [9]. Moreover, various different examples of beneficial interactions between plants and so-called plant growth promoting rhizobacteria (PGPR) are described that are not directly involved in nitrogen fixation. These PGPR either colonize the rhizosphere (the area directly surrounding a root that is influenced by plant root exudates), the surface of plant roots (rhizoplane), or they grow inside roots (endophytic) [10], [11], [12], [13]. Currently, the nature of these interactions is much less understood.

Several mechanisms of growth promotion are discussed [14]. Some PGPR, like *Pseudomonas* spp. support plants to get better access to soil nutrients such as iron or phosphate [15], [16], others, such as *Phyllobacterium* sp. influence the plant's nitrogen metabolism [17]. Furthermore, the production of plant-like hormones by bacteria, such as auxin or gibberellins may affect growth [18], [19], as well as the enzymatic inhibition of plant ethylene synthesis by bacteria [20]. Several soil bacteria are identified to produce volatile compounds that enhance plant growth by unknown mechanisms [21]. Moreover, PGPR can increase tolerance to abiotic stresses, such as salt and drought [22]. Specific bacteria were identified that can have an important impact on plant performance by protecting them against pathogens; either directly via the production of antibiotics, or indirectly by induction of systemic resistance [23].

Many fundamental aspects about the interaction of plants with beneficial bacterial associates are still to be investigated in detail, for example, to what extent (primary) plant metabolism is altered by PGPRs and which transcriptional changes are induced by the beneficial interaction. Research focusing on these interactions has achieved more attention in recent years, for example, whole-genome microarrays were used to obtain insight into the long-term molecular answers of the plant to bacterial colonization [24], [25].

Valuable information about the growth promoting interaction between bacteria and plants was obtained from various studies, in which *Arabidopsis* was inoculated with PGPR isolated from various different crop species [24], [25], [26]. However, it cannot be excluded that mechanisms of plant-bacterial interaction exhibit species-specific characteristics. Some bacteria exert general growth promotion effects in several plant species, other bacteria only do so in interaction with their specific host plant [27]. So far, only a few rhizobacteria are known that are naturally associated with Arabidopsis roots, and none of these show positive growth effects or even act as pathogens (e.g. *Pseudomonas brassicacearum* [28], [29], *P. viridiflava* [30], [31], and *P. thivervalensis MLG45*, a species that elicits induced systemic resistance [32]). To study the complex plant-bacteria interactions that result in direct growth promotion of plants, PGPR that are naturally associated with the model plant Arabidopsis are most valuable. It is likely that specific interactions have been developed during long-term co-evolution, similar to the compatible and incompatible interactions between pathogens and plants [33], [34].

With the aim to identify Arabidopsis specific PGPR, we collected bacteria associated with roots from plants of a natural Arabidopsis population growing in the fields around Golm, NE-Germany. One of the isolates, *Pseudomonas sp.* G62, promotes growth of different Arabidopsis ecotypes under various growth conditions. To our knowledge, this is the first description of a rhizobacterium that is naturally associated with Arabidopsis roots and promotes plant growth directly and independent of induction of disease resistance. Furthermore, using whole genome microarrays, we studied differential gene expression 6 h after plant roots were treated with *Pseudomonas sp.* G62 bacteria. The bacteria induced significant changes in the expression of plant genes encoding gene products involved in primary C and N metabolism and parts of secondary metabolism. Comparison to other microarray experiments revealed that these changes resemble the transcriptional response of plants to carbon starvation. Quantitative real-time RT-PCR revealed that transcriptional changes persist for several weeks. The growth promotion is associated with increased levels of sucrose in roots. These results provide new insight into the molecular and biochemical responses of plants to the colonization by PGPR.

## Results

### *Pseudomonas sp.* G62 was isolated from roots of field-grown Arabidopsis plants and exhibits plant-hormone-related metabolism

Individual colonies were picked from a plate that was inoculated with the supernatant of a crude Arabidopsis Gol-1 extract of surface-sterilized roots. The colonies were repeatedly transferred to new sterile plates until pure cultures were obtained. We got ∼20 different bacterial strains with various colony morphologies. One of these colonies - referred to as strain G62 - was used for the further characterisation described in this study. Sequencing of the 16S rDNA and subsequent comparison using the BLAST tools at http://blast.ncbi.nlm.nih.gov/Blast.cgi and http://greengenes.lbl.gov revealed highest similarity with other sequences derived from the genus Pseudomonas. To determine the 16S rRNA gene sequence, chromosomal DNA was extracted from the bacterial isolate and purified using the PureLink Genomic DNA Mini Kit (Invitrogen) according to the manufacturer's instructions. The unique 16S rDNA sequence of the bacterial strain used in this study can be retrieved from the NCBI GenBank database (http://www.ncbi.nlm.nih.gov/sites/entrez/?db=nucleotide) under the sequence accession number: FN547413. Characterisation of the fatty acid composition and other biochemical/physiological traits (see Table S1) confirmed the classification of strain G62 into the genus Pseudomonas, but further assignment to a specific species was not possible.

*P. sp.* G62 was tested for several traits that are supposed to be associated with plant growth promotion. The strain produces auxin and exhibits activity of ACC deaminase in bacterial growth medium (Table 1). Moreover, *P. sp.* G62 was showing phosphate solubilization activity and produces siderophores (Table 1). The gene *nifH* encoding the enzyme nitrogenase that is required for nitrogen fixation in e.g. *Pseudomonas stutzeri* could not be determined in *P. sp.* G62 (Table 1).

**Table 1:** Properties of *P. sp.* G62: Auxin production (0.9001±0.024 µg IAA/ml), ACC deaminase activity (26.25±5.75 nmol α-ketobutyrate/mg prot * h), phosphate solubilization, siderophore production and presence of the *nifH* (nitrogenase) gene.

Bacteria Property	
Auxin production in liquid medium	**+**
ACC deaminase activity in liquid medium	**+**
Phosphate solubilization	**+**
Siderophore production	**+**
Nitrogenase gene, *nifH*	**−**

### *Pseudomonas sp.* G62 is associated with roots of *Arabidopsis thaliana*

**Figure 1:**
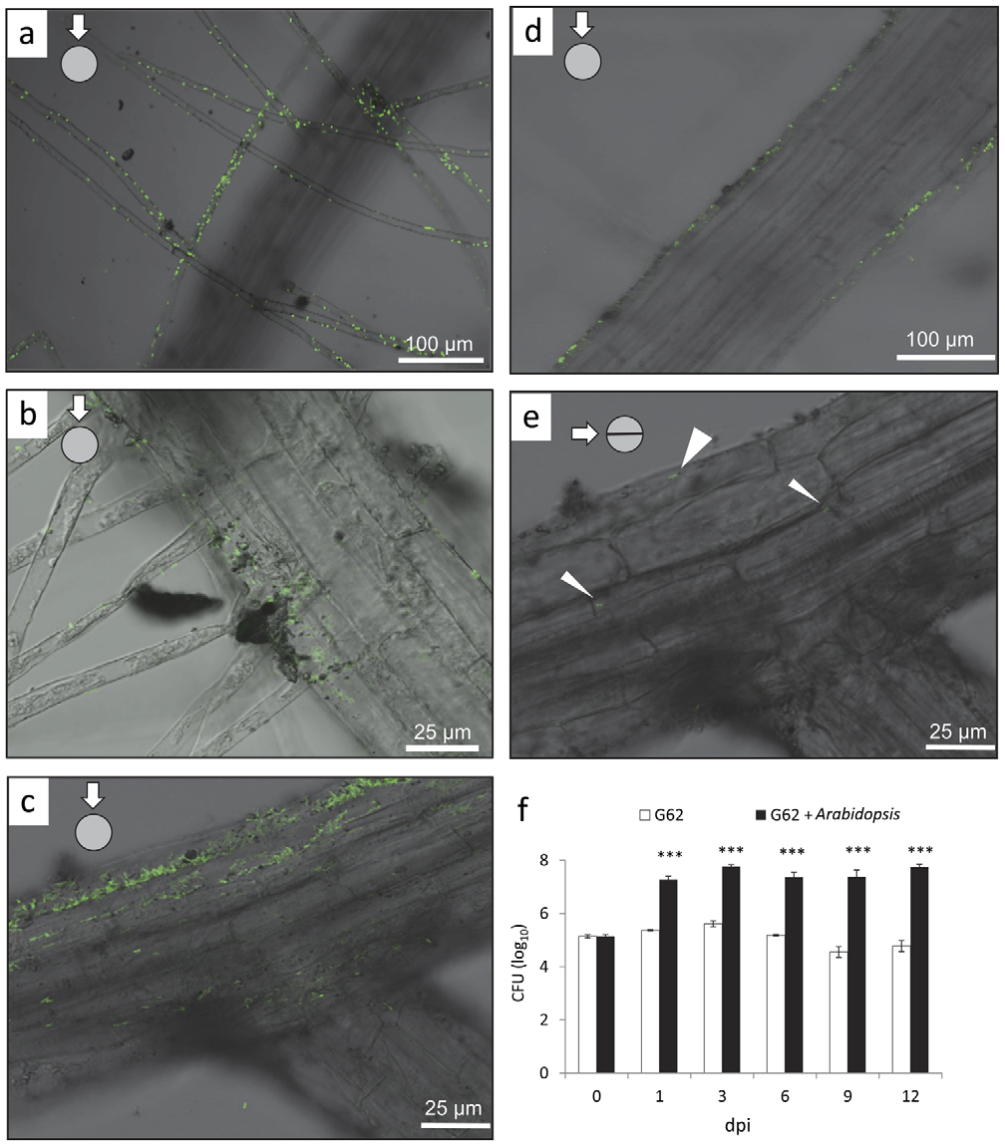
Colonization of *Arabidopsis* (Col-0) roots by *P. sp.* G62 after 3 days, visualized by eGFP - transformed bacteria. a–d: colonization of root hairs and root surface 3 days after inoculation of the root with bacteria, photographs of the root surface. e: sporadic bacteria inside of roots: focus at the middle of the root as can be seen by the focused xylem strand; thin arrows: single bacteria inside of the root, thick arrow: bacterium at root surface. f: cfu (colony forming units) of bacteria in liquid plant medium with or without plants (dpi: days post inoculation). Mean values ± SE, asterisks indicate p-values (***: p<0.001).

The pattern of root colonisation (Col-0) was investigated by using eGFP - transformed *P. sp.* G62 and confocal laser scanning microscopy (CLSM). Bacteria were mainly detected at the rhizoplane and on root hairs (Fig. 1a–d), demonstrating that this species is root associated. Extensive screenings of roots from about 20 plants inoculated with eGFP-carrying bacteria revealed only very few (<10) individual *P. sp.* G62 bacteria inside the roots (Fig. 1e). Despite this sporadic occurrence of endophytically growing individuals, we consider this strain to be an rhizosphere bacterium rather than a true endophyte. No indications were found about lateral transport throughout the entire plant and bacterial occurrence appeared to remain confined to the roots. After 10 weeks of growth, plant roots of agar-medium grown plants were still colonized by 2.1 * 10^8^ cfu per g root, demonstrating efficient long term colonization of the root surface by *P. sp.* G62. Furthermore, growth experiments in a hydroponic culture showed that the amount of bacteria in the medium was 2 to 3 orders of magnitude larger when plants were present, indicating that plant root exudates efficiently support growth of bacteria (Fig. 1f).

### *Pseudomonas sp.* G62 promotes growth and development of *Arabidopsis thaliana*

**Figure 2:**
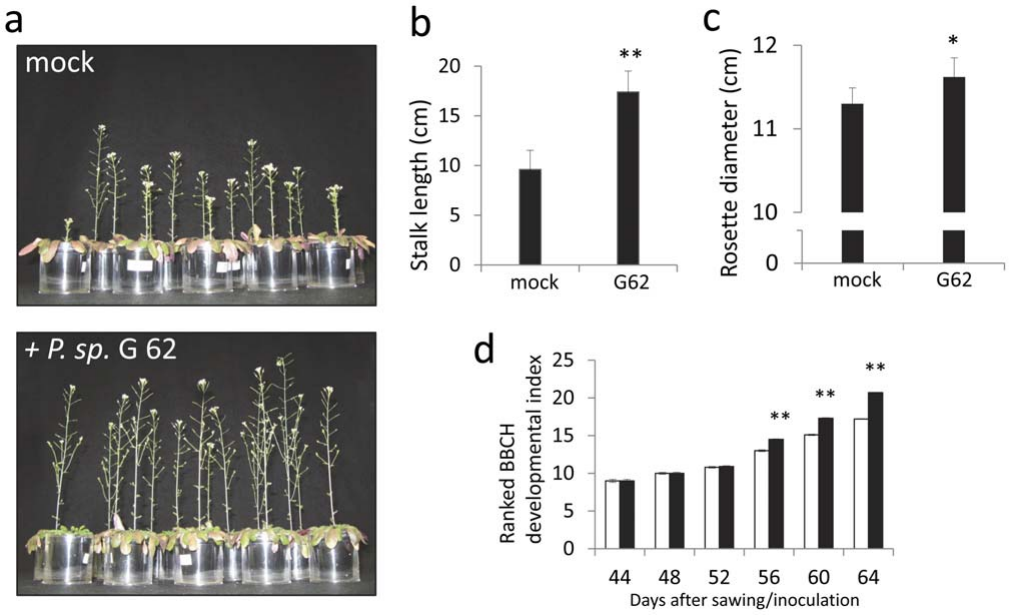
Growth effects of *P. sp.* G62 on *Arabidopsis* ecotype Gol-1 grown in sandy soil. a: Gol-1 with and without bacteria (mock) after a 68-day growth period. b: stalk length. c: rosette diameter. d: developmental index. Mean values ± SE, asterisks indicate p-values (*: p<0.05; **: p<0.01).

**Figure 3:**
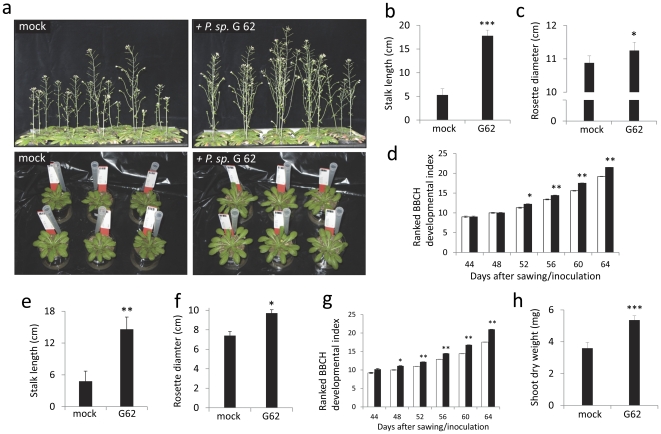
Growth effects of *P. sp.* G62 on *Arabidopsis* ecotype Col-0 on different substrates. a: top panels: Col-0 with and without bacteria (mock) after a 68-day growth period (peat substrate), bottom panels: Col-0 at rosette stage with and without bacteria (mock) grown in sandy soil. b: stalk length. c: rosette diameter. d: developmental index (b–d: grown on peat substrate). e: stalk length. f: rosette diameter. g: developmental index (e–g: grown on sandy soil). h: shoot dry weight (grown in agar medium). Mean values ± SE, asterisks indicate p-values (*: p<0.05; **: p<0.01; ***: p<0.001).

To analyse the effect of bacteria on plant growth, either seeds or the growth substrate were inoculated with *P. sp.* G62. Measurements on rosette diameter and stem length were taken after a two month growth period and the developmental stages of *Arabidopsis* were scored with a stage scale (see Table S1) that was adapted for *A. thaliana* from the published rape seed BBCH-scale and was tested for reproducibility before. Growth promotion was first assayed in the green house using sterilized sandy soil from the Golm field site, where the bacteria and the *Arabidopsis* Gol-1 ecotype, from which the bacteria were originally extracted, naturally occur. The substrate was inoculated with *P. sp.* G62. Stalk length of Gol-1 plants exposed to bacteria was significantly increased by 70% after 64 days (t-test; p<0.01, n = 13–15; Fig. 2a,b), as were rosette diameters (t-test; p<0.05, n = 13–15, Fig. 2c), and plant development progressed faster (t-test; p<0.01, n = 13–15, Fig. 2d). Secondly, growth of Col-0 was assayed on different substrates (peat substrate and plant agar mediumwith high nutrient supply, sandy soil with low nutrient supply). Col-0 seeds were inoculated with *P. sp.* G62 before planting on peat substrate. Again, the bacteria significantly enhanced growth of 7 week old Col-0 plants: stalk length by more than two-fold (t-test; p<0.001, n = 13–15, Fig. 3b), rosette diameter by 3.4% (t-test; p<0.05, n = 13–15, Fig. 3c), as well as development (t-test; p<0.05 or p<0.01, n = 13–15, Fig. 3d).. These growth effects were still visible after about 10 weeks (Fig. 3a, top panels).Moreover, plant growth (stalk length, rosette diameter, development) was significantly increased in sandy soil at low nutrient supply that was inoculated with bacteria before planting seeds (t-test; p<0.01, n = 15–16, Fig. 3e; p<0.05, n = 15–16, Fig. 3f; p<0.05 or p<0.01, n = 15–16, Fig. 3g; Fig. 3a lower panels). Col-0 plants were also grown on vertical agar plates with full nutrition under sterile conditions from seeds that were pre-incubated with *P. sp.* G62 bacteria (for a picture of the plates see Fig. S1). In this experiment, shoot dry weight of 5 week old Col-0 plants was significantly increased (by 49%) by bacteria as compared to control plants that were not exposed to the bacteria (t-test; p<0.001, n = 20–22, Fig. 3h), and root mass was increased by 49.9% by bacteria (0.309±0.0046 mg vs. 0.463±0.0307 mg per root). Together, this demonstrates that *P. sp.* G62 induced growth promotion on two different *Arabidopsis* ecotypes and growth of Col-0 was increased on various substrates with different nutrient conditions. Moreover, growth promotion is efficient under non-sterile and gnotobiotic conditions.

### *Pseudomonas. sp.* G62 alters transcription of genes related to primary metabolism

To investigate the short-term response of *Arabidopsis* to *P. sp.* G62 at the transcriptional level, we evaluated plants with whole genome microarrays (Affymetrix ATH1) in triplicate. Roots of 18-day-old plantlets of Col-0 were incubated with *P. sp.* G62 for 6 h and subsequently, RNA was isolated from the whole seedlings and compared to untreated samples. It was expected that shoot RNA is overrepresented in these samples compared to root RNA, since the roots of plantlets grown in sandy soil were relatively small compared to leaf mass. Seventy-eight genes were significantly (p<0.05, Benjamini-Hochberg correction) up- or down-regulated at least two times (Table S2). Of these changes, the transcript level of 38 genes was upregulated (log_2_-ratio>1) and transcripts of 40 genes were downregulated (log_2_-ratio<−1).

Among the most highly upregulated transcripts were genes related to cell wall metabolism, nitrogen metabolism, sugar transport and senescence. Expressions of the cell wall degrading beta-xylosidases, *BXL1* and *2*, and the cell wall modifying xyloglucan endotransglycosylase 3 (*XTR3*) were upregulated, as well as UDP-glucose epimerase 1 (*UGE1*), which is involved in synthesis of cell wall precursors from UDP-glucose. Glutamate dehydrogenase 2 (*GDH2*) and glutamine-dependent asparagin synthase 1 (*ASN1*), which are involved in amino acid turnover, were upregulated. Furthermore, the sugar-repressed sulfurtransferase senescence 1 (*SEN1*), which is induced by phosphate starvation and pathogens [35], [36], and dark-inducible 10 (*DIN10*) were upregulated, as well as the sugar transporter *STP1*, which mainly represents the sugar transport activity in vegetative tissues of the 14 *STP* genes in *Arabidopsis* [37]. Also, the auxin-responsive genes, *DRM1*, *BT5* and At2g33830 were upregulated. Genes related to regulation of RNA transcription were downregulated, such as circadian clock associated 1 (*CCA1*) and *TOC1*.

### *Pseudomonas sp.* G62-induced transcriptional changes remain stable over several weeks

**Figure 4:**
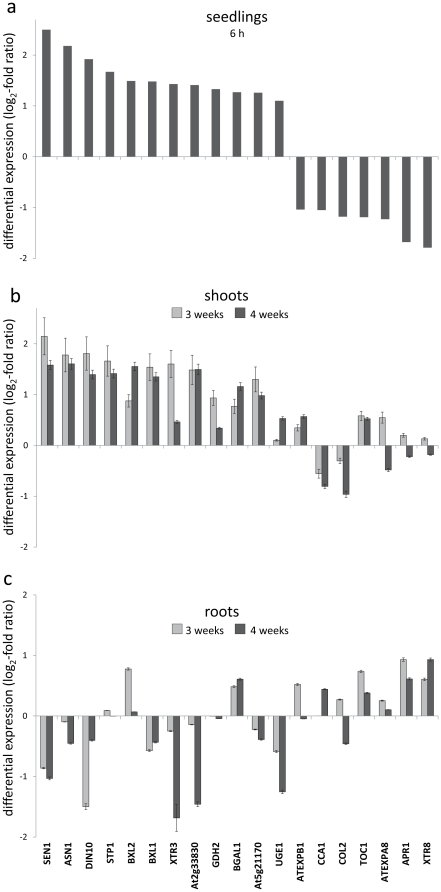
log_2_-fold expression values of *P. sp.* G62 - induced genes. a: selection of the most highly up- or downregulated genes (log_2_–fold change >1 or <−1) determined by microarrays 6 h after bacteria inoculation of 18-day-old seedlings. b, c: expression of the same genes determined by qPCR 3 and 4 weeks after seed inoculation, shoots (b) and roots (c), mean values ± SE.

To evaluate, whether genes that expressed the largest transcriptional changes at the 6 h short-term response remain regulated on a long-term scale, we assessed the transcriptional profile of 19 of the most highly up- or down-regulated genes of the microarray (Tables S2, S3) with quantitative real-time RT-PCR. These genes are associated with carbon-, nitrogen- and cell wall- metabolism and the circadian clock. RNA was separately extracted from shoots and roots of 3 and 4 weeks old plants that underwent seed inoculation with bacteria and were grown on vertical agar plates. Generally, genes that are upregulated 6 h after bacteria treatment are similarly regulated after 3 and 4 weeks in the shoots (Fig. 4a,b), and genes that are downregulated after 6 h show less or no regulation in shoots after 3 and 4 weeks. The sugar-repressed genes *SEN1* (At4g35770) and *DIN10* (At5g20250) are continuously upregulated in shoots. *ASN1* (At3g47340), which is involved in amino acid turnover, and the sugar transporter *STP1* (At1g11260) also remained upregulated. Genes related to the circadian clock and regulation of RNA transcription, such as *CCA1* (At2g46830) and *TOC1* (At5g61380) show attenuated regulation compared to 6 h. The cell wall – associated genes *BXL1* (At5g49360), *BXL2* (At1g02640), *XTR3* (At5g57550) and *BGAL1* (At3g13750) remain upregulated, whereas *EXPB1* (At2g20750), *EXPA8* (At2g40610), and *XTR8* (At3g44990) show reduced regulation compared to the microarrays. In roots (Fig. 4c), the regulation of genes is much less intense than in shoots, and tends to be opposite to shoots. This indicates systemic effects of root colonization by *P. sp.* G62 that influence transcription of genes related to different metabolic pathways in shoots.

### *Pseudomonas sp.* G62-induced transcriptional changes predominantly overlap with sugar starvation

The expression of the genes that were significantly up- or down-regulated after 6 h by *P. sp.* G62 (327 genes with a change 1.5-fold up or down, see Table S5) was surveyed across all approximately 300 different *Arabidopsis* microarray experiments that can be retrieved with the Genevestigator online-tool [38]. The expressional patterns were clustered using the TM4 software tool [39] and the output is shown in Figure S2. The comparison revealed highest similarity between the gene regulation caused by inoculation with *P. sp.* G62 and conditions that induce sugar depletion such as low CO_2_ and extended night [40], [41]. Moreover, experiments that supply glucose and sucrose caused opposite transcriptional responses as compared to inoculation with *P. sp.* G62.

**Figure 5:**
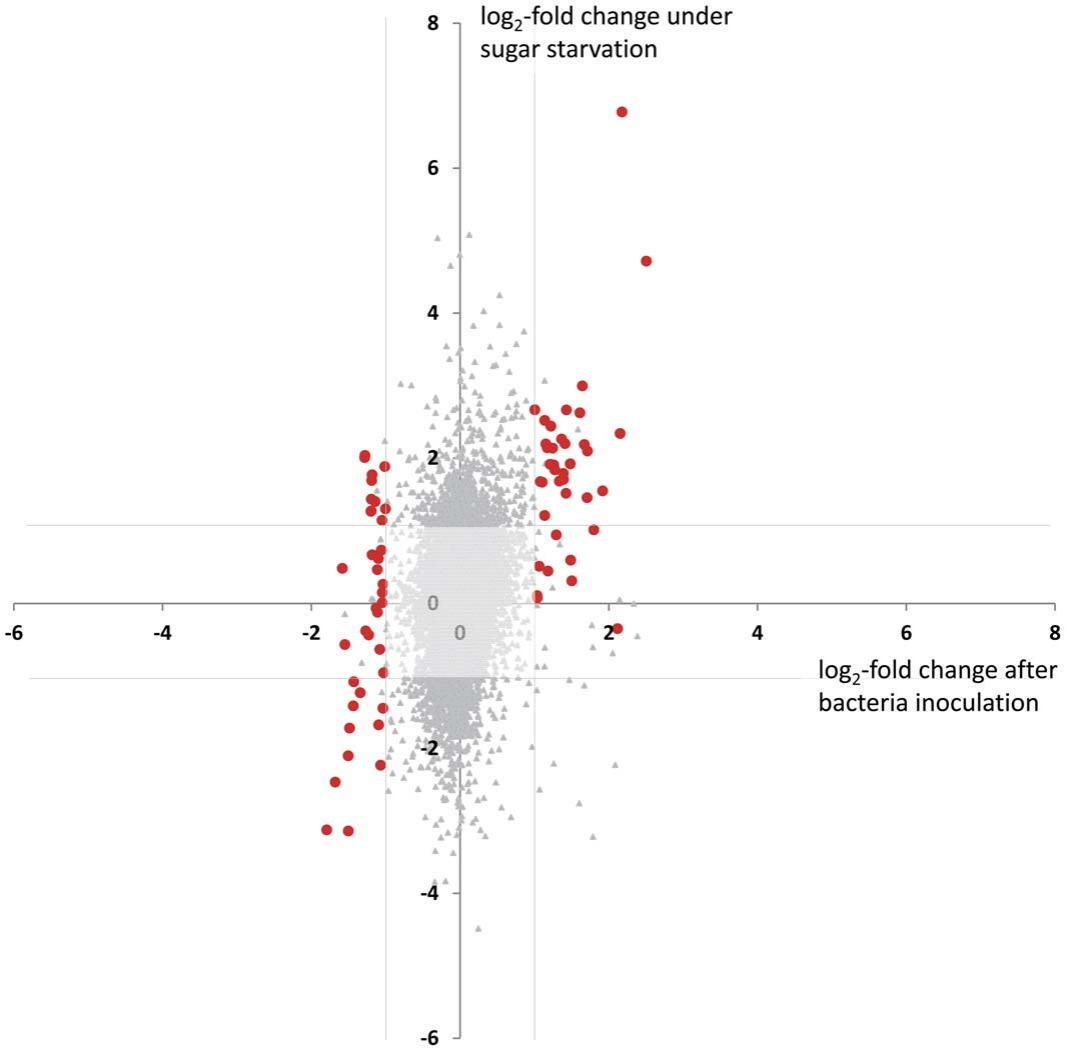
Comparison of log_2_-fold expression values of genes between a 6 h *P. sp.* G62 inoculation (x-axis) and 3 h of sugar starvation (y-axis, [**42**] **).** Genes marked in red are significantly regulated by bacteria (Table 1) with a log_2_-fold change <−1 or >1. Lines parallel to the axes indicate log_2_-fold change of 1 or −1.

As *P. sp.* G62 changed expression of genes for primary carbohydrate metabolism and upregulated sugar-repressed genes, we compared our microarray data specifically with a study on the impact of sugar starvation on gene expression [42](Fig. 5). This study compared three-week-old *Arabidopsis* Col-0 seedlings grown in liquid cultures under low light supplied either with full nutrient medium including 15 mM sucrose or full nutrient medium without sucrose for several days. As in our study, whole seedlings were sampled for microarrays. Comparison of gene expression revealed that genes, which are signifcantly regulated by bacteria with a log_2_-fold change <−1 or >1, clearly correlate with regulation by sugar starvation (Fig. 5). A few genes that are downregulated by bacteria are upregulated by sugar starvation (see Table S6), demonstrating specific effects of bacterial signaling that differ from induction of sugar starvation.

Apart from the selection of individual genes that are significantly up- or downregulated, it was also tested if the expression of specific groups of genes that are related to a common functional category behave differently from what would be expected based on the global expression behaviour of all genes on the array. For this, all genes were ordered in so-called functional categories that contain genes that are attributed to a distinct pathway or function, such as ‘sucrose synthesis’ or ‘abscisic acid-responsive’, according to the annotation criteria described for the MapMan software [43]. This calculation reveals which physiological functions are especially affected by the interaction between *Arabidopsis* and *P. sp.* G62 even when the actual fold change of single genes is relatively small. Using the Wilcoxon Rank Sum test after Benjamini Hochberg correction, the probability was calculated if the distribution of transcript abundance as determined for the genes from one functional category resembles the global expression pattern of all other genes on the entire array. Thus, a low p-value indicates that the expression profile of a sub-population of transcript levels is more likely to be different from the general changes in gene expression.

**Figure 6:**
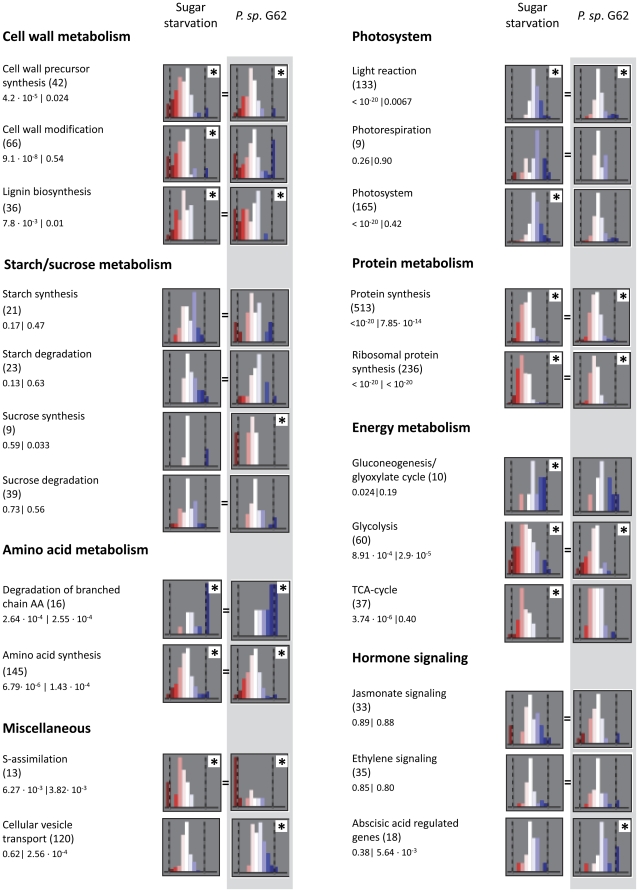
Comparison of the regulation of functional gene categories according to MapMan [**43**] between sugar starvation and *P. sp.* G62 inoculation. Histograms show number of genes on the y-axis and log_2_-FC-categories on the x-axis. Number of genes per functional category is given in brackets, followed by the respective p-values for the categories.

In the presence of *P. sp.* G62, the transcript level changed for genes attributed to processes of primary and secondary metabolism. (Fig. 6). Expressional behaviour of genes involved in sucrose synthesis and glycolysis showed a category-specific tendency to decreased transcript levels after inoculation with *P. sp.* G62, whereas photosynthetic light reaction genes were particularly upregulated. Cell wall precursor synthesis and cell wall modification were generally downregulated, although some genes of these functional categories were significantly upregulated, such as *UGE1* and *XTR3* (Table S2), indicating a specific differential regulation of these genes. Further downregulation was found in protein- and amino acid synthesis. The significant upregulation of genes related to degradation of branched amino acids (Val, Leu, Ile), including methylcrotonoyl-CoA carboxylase (Table S2) indicates the activation of leucine catabolism, which can serve as energy source under sugar starvation [44]. Photosynthesis genes, cell wall, protein and amino acid metabolism genes were regulated similarly between sugar starvation and *P. sp.* G62 inoculation, but genes related to sucrose metabolism were not affected by sugar starvation. Genes responsible for cell wall precursors were downregulated in both experiments, with the exception of two UDP-glucose epimerases, one of which was also upregulated in our study (Table S2). A number of genes of the metabolic category for cell wall metabolism, including expansin-like A1, B1 and A8, as well as XTR 3, 7 and 8, were similarly regulated, and the energy- and carbon-demanding sulfur assimilation was downregulated in both experiments. TCA cycle genes, in contrast, were significantly downregulated by sugar starvation, but not affected by *P. sp.* G62. Furthermore, important genes required for breakdown of sucrose were differentially regulated between *P. sp.* G62 treatment and sugar starvation. Under sugar starvation, a cytosolic invertase was upregulated, whereas the bacteria slightly induced sucrose synthase that catabolizes sucrose. Regulation of hormone signaling was different for abscisic acid regulated genes, which were affected by the bacteria. Together, these data indicate a partial overlap of differential gene expression induced by *P. sp.* G62 and sugar starvation, despite differences in parts of primary metabolism.

### *Pseudomonas sp.* G62 induced a long-term change of sucrose content in roots

**Figure 7:**
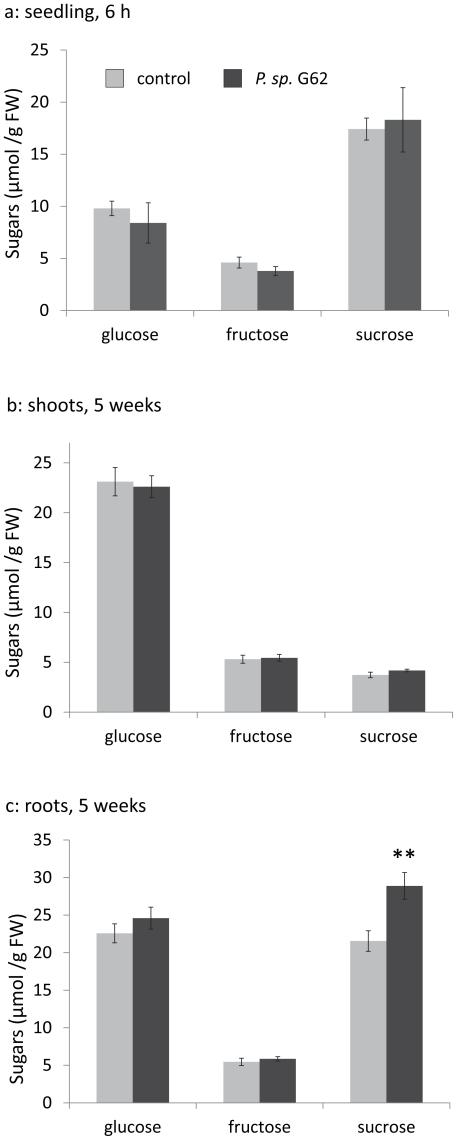
Levels of sucrose, glucose and fructose in different tissues after bacteria treatment. a: sugars in seedlings treated with bacteria for 6 h; sugars in shoots (b) and roots (c) of 5-week-old *Arabidopsis* Col-0 plants after seed inoculation with *P. sp.* G62. Mean values ± SE, asterisks indicate p-values (**: p<0.01).

The sugar status (sucrose, fructose, glucose) of 18-day-old plants that were treated with bacteria for 6 h did not change (Fig. 7a), even though the transcriptional profile already showed similarity to sugar starvation (Figs. 5, 6, Fig. S2) and sugar-repressed genes such as *SEN1*, *DIN10* and *STP1* were induced (Table S2, Fig. 4a). To study whether the increased growth after 5 weeks (Fig. 3) is reflected in the carbohydrate and protein status of the plants, we measured levels of glucose, fructose, sucrose and starch levels as well as the amount of soluble protein and free amino acids in leaves and roots of agar-medium grown 5-week old Col-0 plants, of which the seeds have been treated with *P. sp.* G62. Sucrose levels were increased by 34% in roots of bacteria-treated plants as compared to roots of non-inoculated plants (Fig. 7c, t-test, p<0.01, n = 8–10), whereas the other metabolites that were analyzed did not alter upon *P. sp.* G62 colonization (data not shown).

## Discussion

Understanding the mechanisms underlying the growth promoting effects of rhizobacteria is important to design strategies for their application in agriculture, such as biofertilization, biocontrol and phytoremediation [45], [46]. The beneficial effects of PGPR are attributed to several mechanisms, such as enhanced efficiency of the plant's nutrient uptake [47], hormone production [48], or, indirectly, to defense against pathogens [49]. However, the molecular events underlying plant growth promotion by PGPR are still poorly understood. Here, we studied short- and long-term effects of an *Arabidopsis*-associated rhizobacterium on gene transcription as well as on primary metabolites and growth.

The growth-promoting rhizobacterium, *P. sp.* G62, was extracted from roots of field-grown *Arabidopsis* plants. Genetically modified strains of *P. sp.* G62 expressing fluorescent protein were mainly localized on the surface of roots and root hairs and only very rarely an individual bacterium was observed in root interior (Fig. 1). *P. sp.* G62 is capable of significantly promoting plant growth with respect to rosette diameter, stalk elongation, development and biomass under high and low nutrient conditions (Fig. 3). It is unlikely that the observed effects are related to bacteria-mediated improvement of nutrient uptake rather than resulting from other mechanisms of growth promotion because similar growth effects were observed on three different media (sandy soil, peat substrate or ½ M&S agar medium) with either high or low nutrient quality. Even though it is assumed that plant growth-promoting bacteria typically have little or no measurable effect on plant growth when the plants are cultivated under optimal and stress-free conditions [20], *P. sp.* G62 stimulated plant growth even under our various favorable nutrient supply and growth conditions. Probably, bacterial production of auxin or ACC-deaminase activity (that may reduce ethylene production by the roots) is involved in the observed stimulation of plant growth. Moreover, G62 was found to be able to solubilize phosphate or increase iron availability via siderophore production (Table 1). However, since growth promotion was also promoted by G62 when neither phosphate nor iron availability was limiting for the plant, it is assumed to be unlikely that these effects are responsible for the growth effects of the plants.

Various plant-microbe interactions were previously described to have a strong effect on plant carbon metabolism [50], [51]. This may be due to the attempt of the bacteria to manipulate plant metabolism in order to get access to nutrients, but may also be linked to positive growth effects of bacteria on the plants. To investigate whether a modification of primary metabolism by *P. sp.* G62 is already manifested on the transcriptional level at an early stage of plant-bacteria interaction, we used Affymetrix microarrays to determine differential gene expression 6 h after plant inoculation. Compared to control plants, 78 genes were at least two times up- or downregulated (Tab. 1). A clear bias towards differential expression of groups of genes was observed that are involved in the same physiological or biochemical processes, such as glycolysis, sucrose synthesis, amino acid, protein and cell wall metabolism (Fig. 6).

Interestingly, comparison of our data with 300 publically available microarray analyses revealed that the gene expression profile obtained after inoculation with *P. sp.* G62 largely resembled transcriptional reorganization during sugar (sucrose) starvation (Figs. 5, 6, Figure S2). A detailed comparison between our data and the transcriptional changes induced by sucrose starvation as described by [42] revealed that especially genes related to carbohydrate metabolism were similarly regulated, such as the categories of photosynthesis (up), glycolysis (down) and cell wall precursor synthesis (down) (Fig. 6). In contrast, the sucrose synthesis category was only downregulated after bacteria treatment, and the TCA cycle and cell wall metabolism categories were only downregulated by sugar starvation. Apparently, effects of sugar starvation have a more pronounced effect on genes related to sugar metabolism than bacteria. This can be recognized from reduced expression of genes related to mitochondrial respiration (TCA cycle) and a larger activation of genes related to photosynthesis by sugar starvation (Fig. 6). Nineteen of the most strongly regulated genes of the microarray, which are mainly related to energy and cell wall metabolism, showed a stable expression pattern during 4 weeks in shoots (Fig. 4b), indicating a continuous effect on primary metabolism by bacteria.

The gene expression analysis shows that *P. sp.* G62 bacteria rapidly induced a set of transcriptional responses in the plant that are supposed to support a plant during carbohydrate starvation. However, plant sugar levels were unchanged 6 h after bacteria inoculation (Fig. 7a). Moreover, 5 weeks after bacteria inoculation, when positive growth effects were already manifested, sugar levels were unchanged (glucose and fructose) or even increased (sucrose), demonstrating that the plants are not limited in carbohydrate supplies (Figs. 2, 7). Apparently, the plant provides more sucrose to roots when bacteria are present. It is conceivable to assume that the increased carbohydrate status benefits growth of root-associated bacteria when carbon increasingly exudes into the rhizosphere. Such leakage of carbohydrates can represent a considerable amount of assimilated carbon of *Arabidopsis* and serves as nutrients for rhizosphere bacteria [52], [53]. Indeed, *P. sp.* G62 grew far better in liquid *Arabidopsis* growth medium in the presence of plant roots (exudates) than without plants (Fig. 1f).

Hence, it may be speculated that the bacteria via yet unknown signaling effectors mimic a state of sugar starvation in the plant, which is uncoupled of its true carbohydrate status. The increased net availability of soluble carbohydrates may not only benefit bacteria at the rhizoplane, but may also promote growth of the plant host. For example, the increased amount of sucrose may serve as energy source and as primary substrate for the synthesis of cell wall components that are required during growth. The increased dry weight per plant after inoculation with *P. sp.* G62 (Fig. 3h) suggests that the loss of carbon to the rhizosphere is compensated for by an increased gross photosynthetic carbon fixation that is probably due to the increased leaf area of the plants. It should be noted however, that in contrast to the stimulation of photosynthetic capacity that was, for example, observed after inoculation with the soil bacterium *Bacillus subtilis* GB03 [54], no increase in photosynthetic activity expressed per unit of leave area was observed in our system (Fig. S3).

In summary, we demonstrated that the bacterial strain *P. sp.* G62 that was extracted from roots of the wild *Arabidopsis* ecotype Gol-1 promoted vegetative growth of the *Arabidopsis* ecotypes Gol-1 and Col-0. This can be recognized by increased rosette diameter, stalk elongation, accelerated development and increased biomass (Figs. 2, 3). Growth was promoted on nutrient-rich peat substrate, nutrient-poor sandy soil and on sterile agar plates with M&S medium. This indicates that the growth effect is not simply explained by just resolving (micro)nutrient depletion. Early interaction of *Arabidopsis* and *P. sp.* G62 resulted in a regulation of transcripts of primary metabolism that is partially similar to the response to carbohydrate starvation and points to increased metabolic demand for sugars and energy; however, soluble sugars are not reduced after 6 h. The transcriptional response of upregulated genes is stable in shoots during 4 weeks, indicating that bacterial effects are systemic and constant over time. The bacteria did not alter protein and amino acid content of 5 week old plants, but induced an increase of sucrose levels in roots to levels higher than in non-inoculated plants. This may both serve to promote plant growth and act as a precursor for nutrients that the plant offers to its beneficial resident. The signal by which the bacteria induce the differential gene expression in the plant remains unknown. Since host-specific effects of bacteria are likely to differ from general effects that can be found also on non-host species, the characterization of growth-promoting bacteria that are naturally associated with *Arabidopsis* is highly valuable for further research.

## Materials and Methods

### Collection and determination of bacteria

Field grown *Arabidopsis thaliana* ecotype Gol-1 plants [local ecotype found in Golm, Berlin area, Germany, characterized and genotyped in [55]] were collected from a field on sandy soil in Golm (52°24′57″N, 12°58′18″E, Potsdam, Germany). Roots were surface-sterilized with 70% ethanol for 1 min and 2.5% sodium hypochloride for 3 min and rinsed three times in sterile distilled water. Several roots were homogenised and incubated in ¼ strength Ringer's solution for 45 min on a rotary shaker. Dilution series of the supernantant of this suspensions were spread on agar plates with tryptic soybean medium (TSA). Individual colonies were picked after incubation at 16°C for one to three days. After the initial selection, single bacteria colonies were repeatedly transferred to new TSA plates until cultures appeared to be pure.

The 16S rRNA gene sequence (1470 bp) was amplified by PCR with the universal primers P0 and P6 as described [56] and the Advantage HF2 PCR kit (Clontech). Purified PCR products were sequenced by AGOWA (Berlin, Germany).

### Bacteria properties

Activity of ACC deaminase (AcdS) was measured as described by [57]. Briefly, bacteria were grown in ACC supplied minimal medium and production of α-ketobutyrate was measured photometrically (530 nm). Measurements were normalized against a standard curve of α-ketobutyrate. Protein content of bacteria was determined with the bicinchoninic acid assay [58]. Measurements were carried out three times with at least 4 biological replicates. Production of IAA in tryptophane-enriched TSB medium was determined colorimetrically as described by [59] and normalized to bacterial OD_600_. Measurements were carried out three times with at least 4 biological replicates. Phosphate solubilization was measured on medium amended with insoluble calcium phosphate according to [60]. Production of siderophores was estimated with the chrome azurol S assay according to [61]. Detection of the *nifH* gene that encodes a nitrogenase was carried out according to [62] by nested PCR on bacterial chromosomal DNA with degenerated universal *nifH* primers. As a positive control we used DNA of the strain *P. stutzeri* that was obtained from the German Collection of Microorganisms and Cell Cultures (DSMZ, Braunschweig, Germany; DSM number 4166).

### Plant and bacteria growth conditions

Seeds of two *Arabidopsis* ecotypes Columbia (Col-0) and Gol-1 were used for growth on agar plates, peat substrate (made of peat, vermiculite and sand) with full nutrient supply (MPI *Arabidopsis* substrate, Stender, Germany) or sandy soil. Sandy soil was gained from the A layer of a field site in Golm. This soil layer was previously found to be limited in magnesium and nitrogen, but rich in phosphate (nutrient composition: 3 kg ammonium-N/ha, 8 kg nitrate-N/ha, 11 kg total N/ha, 84 mg P/kg, 110 mg K/kg, 29 mg Mg/kg). This soil was autoclaved twice and then stored for three months to re-establish ion balance.

Vertical agar plates were prepared by filling square petri dishes with side length of 15 cm up to three quarters with ½ M&S medium [63]. Seedlings on agar plates were grown for 5–7 weeks. Before planting, seeds were first sterilized and then incubated for 4 to 6 h in 10 mM MgSO_4_ (mock) or in a bacteria solution. Bacteria were grown in an overnight liquid culture (tryptic soybean medium), centrifuged and resuspended in 10 mM MgSO_4_ to an OD_600_ of 1 (5.1*10^8^ CFU/ml). Plants in a climate chamber were grown with 12 h day (22°C, relative humidity 60%) and 12 h night (20°C, relative humidity 75%) at 250 µE/m^2^. To initially establish optimal humidity for germination of seeds grown on sandy soil, 50 ml of dry sandy soil was inoculated with 25 ml bacteria solution or mock solution (10 mM MgSO_4_). Sterilized seeds (EtOH and sodium hypochloride treatment) were then planted on the substrate. Plant development was scored based on a ranked developmental stage scale that was developed based on the BBCH scale for canola [64] that was modified for *Arabidopsis* (see Table S3). To determine cfu of *P. sp.* G62 of 12-week old plant roots, roots of agar-medium grown plants were washed with 5 ml TSB medium. LB plates amended with 100 µg/ml ampicilin and 100 µg/ml cycloheximide were used to grow dilutions of *P. sp.* G62.

For determination of bacterial growth in the presence of plants, in a 24 well plate 1 ml of ½ M&S liquid medium was inoculated with bacteria to an OD_600_ of 0.4. In half of the wells, one *Arabidopsis* plant (14 days old) per well was grown hydroponically. CFU of bacteria were determined for 12 days after inoculation by plating of serial dilutions on TSA medium. The experiment was replicated three times with similar results.

### Transformation of bacteria with eGFP

*Pseudomonas* sp. G62 was transformed with the *eGFP*-carrying plasmid pMP4655 [65] by triparental mating. The wild type strain was resistant to ampicilin (40 µg/ml), chloramphenicol (20 µg/ml) and cycloheximide (100 µg/ml), but was sensitive to tetracycline (80 µg/ml) and kanamycin (50 µg/ml). *E. coli* DH5α containing pMP4655 [66] was used as donor strain and *E. coli* K12 HB101 harboring the plasmid pRK2013 as helper strain, which was obtained from the German Collection of Microorganisms and Cell Cultures (DSMZ, Braunschweig, Germany). Transformed bacteria containing the plasmid pMP4655 that codes for the enhanced GFP protein were selected on LB agar plates supplemented with 80 µg/ml tetracycline. Fluorescent bacteria were identified under UV light.

### Confocal laser scanning microscopy analysis of root colonization by *Pseudomonas* sp. G62

In order to localize bacteria on *Arabidopsis* roots, 7 days old seedlings were transferred to sandy soil containing eGFP expressing bacteria and after 3 days of subsequent growth root samples were taken and rinsed with water. The localization of bacteria was examined with a confocal laser scanning microscope (Leica TCS SP5, Wetzlar, Germany) equipped with an argon laser. eGFP was excited at 470 nm and detected at 525 nm.

### Microarray analysis

One or two Col-0 seedlings were grown in 1.5 ml Eppendorf tubes, from which the bottom was cut off. The tubes contained sandy soil and were placed on peat substrate. Six hours before harvesting 18-day old seedlings, 500 µl of *P. sp.* G62 (OD 0.5 in 10 mM MgSO_4_), or MgSO_4_ without bacteria (mock) for controls were applied to the medium. Twenty-five seedlings were pooled for each of three replicates per treatment. RNA was extracted from whole seedlings with RNeasy Mini Kit from Quiagen according to the manufacturer's protocol. RNA quality check and microarray hybridization was carried out by Atlas Biolabs (Berlin, Germany). Chip quality was analysed and raw data (.cel files) were evaluated and normalised with the ROBIN software (criteria: robust multi array averaging, nested F-test, Benjamini-Hochberg correction) [67]. The orignal data can be downloaded from the GEO database (http://www.ncbi.nlm.nih.gov/geo/) with the GEO accession GSE24552. Microarrays were evaluated with ROBIN, the Genevestigator online-tool [38] and visualized with the TM4 software tool [39]. Microarray comparison was further carried out with the MapMan tool [43].

### RT-qPCR

Total RNA was extracted from 3 and 4 week old plants that were continuously colonized by bacteria and grew in vertical agar plates (control plants were not inoculated). Five plants were pooled for each of the 5 replicates per treatment. RNA was extracted using the RNeasy kit (Qiagen, http://www.qiagen.com/) for shoots and using the InviTrap Spin Plant RNA Mini Kit (Invitek, http://www.stratec-biomedical.de/) for roots. RNA was subjected to DNase treatment using the TURBO DNA-free kit (Ambion, http://www.ambion.com/). Five micrograms of RNA were reverse transcribed into cDNA using the Superscript III reverse transcriptase kit (Invitrogen). RT-qPCR amplification was carried out with the ABI Prism 7900 sequence detection system (Applied Biosystems, http://www.appliedbiosystems.com/), using a power SYBR green master mix (Applied Biosystems) and the primers as described in Table S4. UBQ10 (At4g05320), Pex 4 (At5g25760), bHLH (At4g38070), SAND (At2g28390) and PP2A (At1g13320) were used as reference genes [68](Table S4). Relative quantification of the expression of each individual gene was performed using the comparative threshold cycle method, as described in the ABI PRISM 7900 sequence detection system user bulletin no. 2 (Applied Biosystems). Primer efficiencies were calculated with the LinReg software (http://LinRegPCR.HFRC.nl).

### Metabolite measurements

All measurements were performed on two independent experiments. Seeds were inoculated in a bacterial suspension (OD_600_ 0.5) and grown for five weeks on vertical agar plates supplied with 1/2 M&S, Plants treated with bacteria for 6 h were grown as described for the microarray experiment. Plants were harvested in the middle of the light phase (2 pm). Each treatment was measured with 8–10 replicates, with 4–5 plants pooled for one replicate. Sucrose, glucose and fructose were assayed in EtOH according to [69], starch was measured according to the UV-method from Roche (Mannheim, Germany), amino acids were measured according to [70], and protein was measured with Nanoquant (Roth, Karlsruhe, Germany).

### Statistical analysis

Statistical analysis was carried out with SigmaPlot software version 11.0 from Systat Software, San Jose, USA.

### Ethics statement

The wild *Arabidopsis* plants from which bacteria were extracted were taken with permission from the Max Planck Society from land that is privately owned and protected by the Max Planck Society. *Arabidopsis* is not an endangered or protected species.

## Supporting Information

Figure S1 ***Arabidopsis*** **Col-0 plants 4 weeks after seed inoculation with** ***P. sp.*** **G62, grown in vertical plates with ½ M&S medium.**
(TIF)

Figure S2 **Comparison of** ***P. sp.*** **G62-regulated genes across 300 different microarray experiments.**
(TIF)

Figure S3 **Comparison of photosynthetic activity of 33-day old Col-0 plants growing in peat substrate with and without bacteria.**
(TIF)

Table S1 **Features of** ***P. sp.*** **G62.**
(TIF)

Table S2 **List of genes that are differentially regulated in whole seedlings of** ***A. thaliana*** **by** ***P. sp.*** **G62 6 h after root inoculation compared with mock treated plants (log_2_-fold change, adjusted p-value).**
(DOCX)

Table S3 **BBCH scale for evaluation of plant development.**
(DOCX)

Table S4 **Genes and primers used for qPCR.**
(XLSX)

Table S5 **List of genes that are significantly regulated (>1.5 or <−1.5 fold) by** ***P. sp.*** **G62.**
(XLSX)

Table S6 **List of genes that are down-regulated by** ***P. sp.*** **G62, but upregulated by sugar starvation.**
(DOCX)
