# Supplementary figures and images for: A Naturally Associated Rhizobacterium of Arabidopsis thaliana Induces a Starvation-Like Transcriptional Response while Promoting Growth
Source: PLoS One. 2011 Dec 28;6(12):e29382. doi: 10.1371/journal.pone.0029382 (PMC3247267; doi:10.1371/journal.pone.0029382)

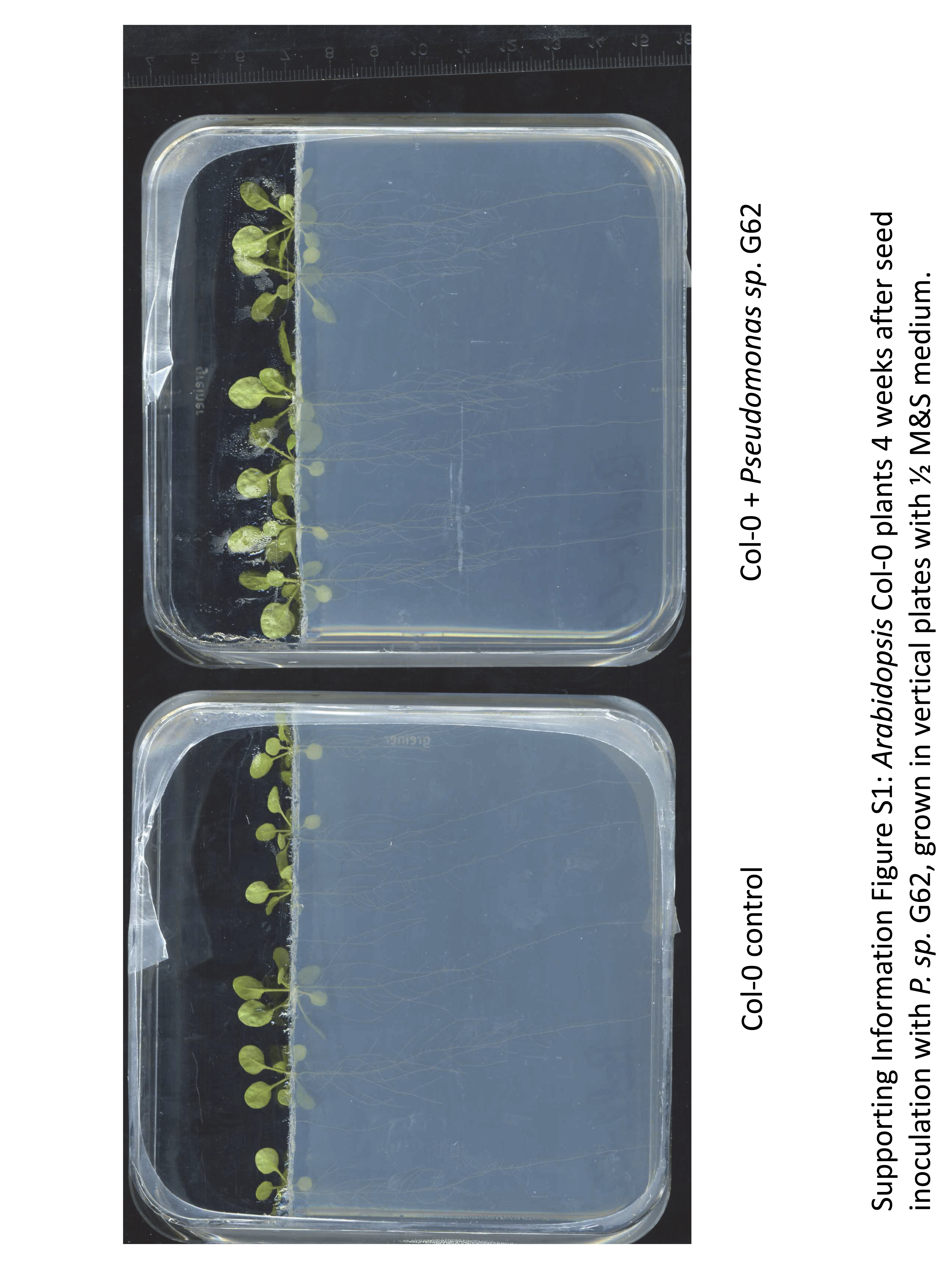

Supplement: Figure S1 — Arabidopsis Col-0 plants 4 weeks after seed inoculation with P. sp. G62, grown in vertical plates with ½ M&S medium. (TIF) [file pone.0029382.s001.tif]

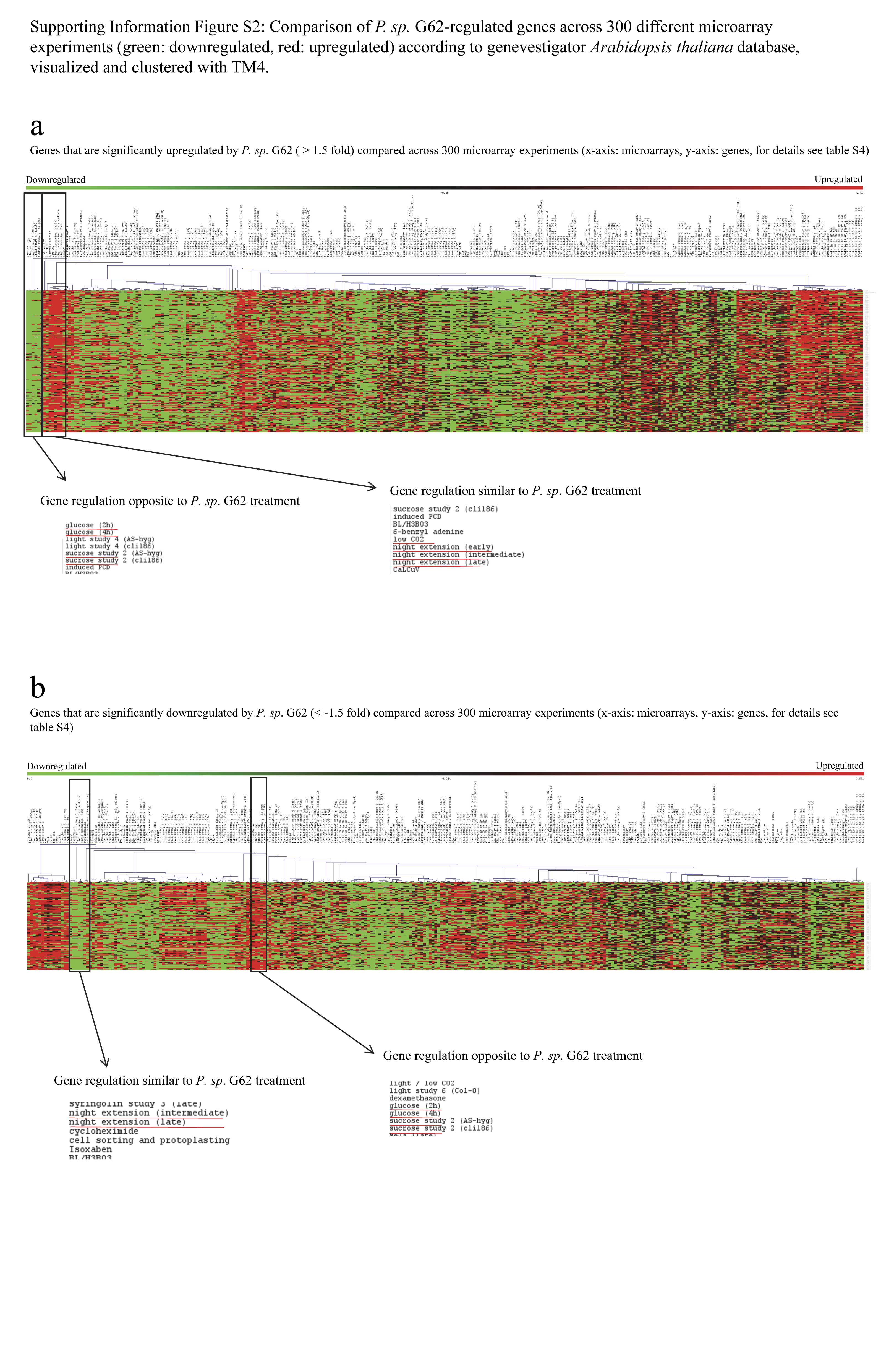

Supplement: Figure S2 — Comparison of P. sp. G62-regulated genes across 300 different microarray experiments. (TIF) [file pone.0029382.s002.tif]

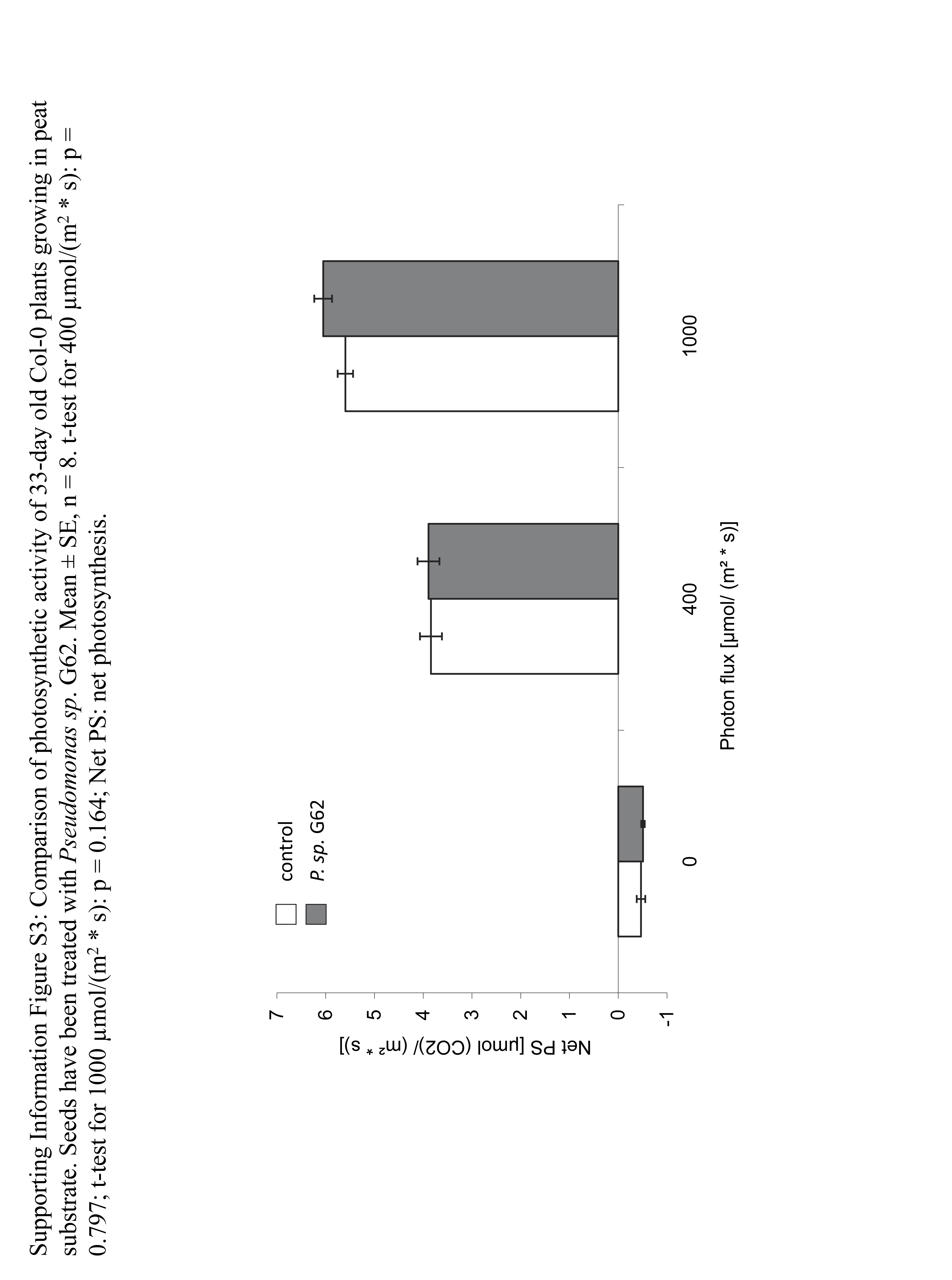

Supplement: Figure S3 — Comparison of photosynthetic activity of 33-day old Col-0 plants growing in peat substrate with and without bacteria. (TIF) [file pone.0029382.s003.tif]

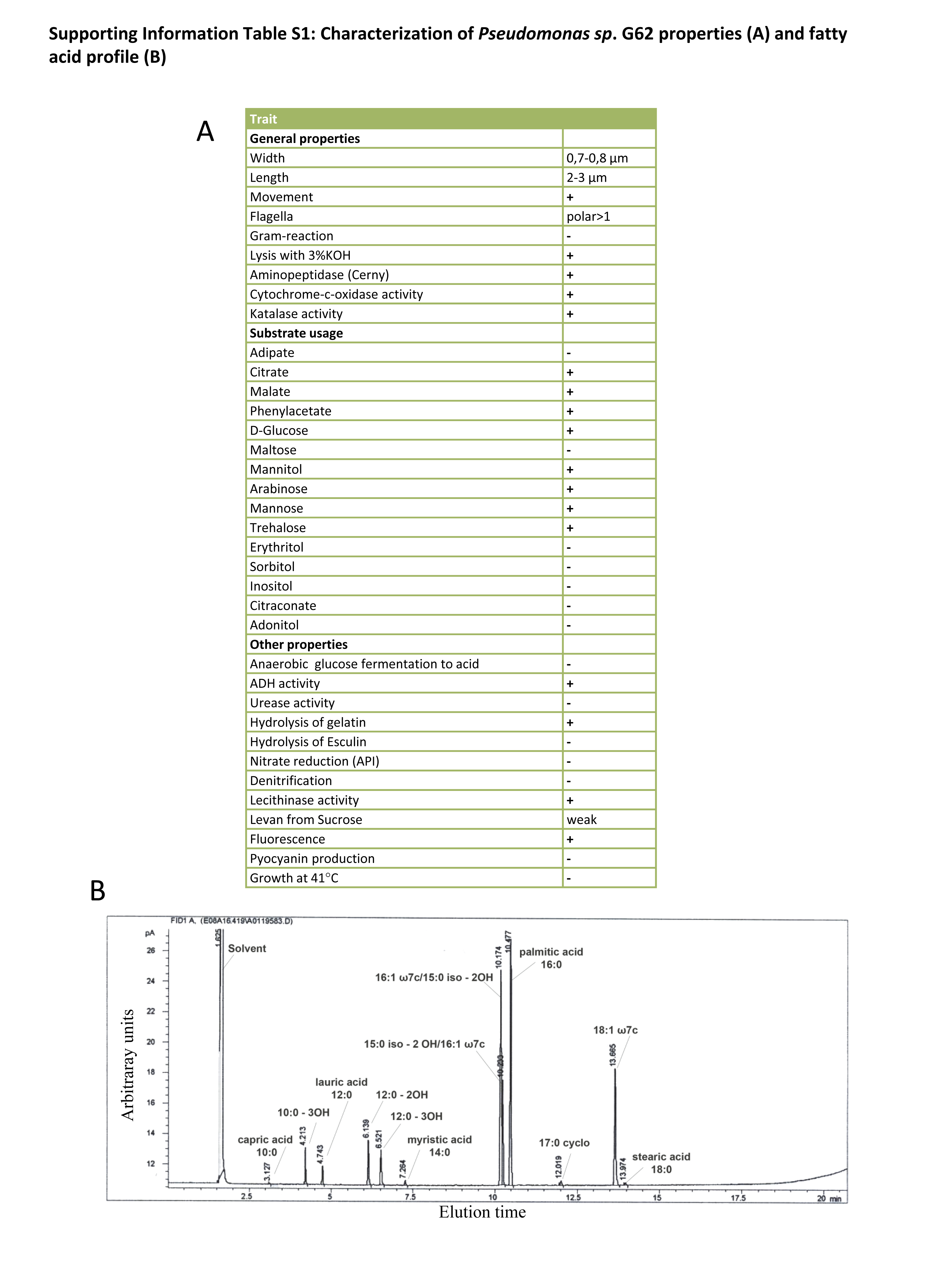

Supplement: Table S1 — Features of P. sp. G62. (TIF) [file pone.0029382.s004.tif]
