# Supplementary material for: A Naturally Associated Rhizobacterium of Arabidopsis thaliana Induces a Starvation-Like Transcriptional Response while Promoting Growth
Source: PLoS One. 2011 Dec 28;6(12):e29382. doi: 10.1371/journal.pone.0029382 (PMC3247267; doi:10.1371/journal.pone.0029382)
Supplement: Table S2 — List of genes that are differentially regulated in whole seedlings of A. thaliana by P. sp. G62 6 h after root inoculation compared with mock treated plants (log2-fold change, adjusted p-value). (DOCX) [file pone.0029382.s005.docx]

Supporting Information Table S2: List of genes that are differentially regulated in whole seedlings of *A. thaliana* by *P. sp*. G62 6 h after root inoculation compared with mock treated plants (log_2_-fold change, adjusted p-value). Genes are ordered in groups of functional categories according to MapMan (Usadel B, Nagel A, Thimm O, Redestig H, Blaesing OE, et al. 2005. Extension of the visualization tool MapMan to allow statistical analysis of arrays, display of corresponding genes, and comparison with known responses. Plant Physiology 138: 1195-1204)

| MapMan category/Gene annotation | log_2_-fold change | adj. p-value | Gene ID |
| --- | --- | --- | --- |
| **3.1 minor CHO-metabolism.raffinose-family** |  |  |  |
| DIN10 (Dark inducible 10) | 1,92 | 1.42E-02 | At5g20250 |
| AtSIP2 (Arabidopsis thaliana seed imbibition 2) | 1,14 | 8.93E-03 | At3g57520 |
| **9.8 mitochondrial electron transport/ ATP-synthesis.uncoupling protein** |  |  |  |
| Mitochondrial substrate carrier family-Protein | -1,05 | 7.41E-03 | At4g24570 |
| **10.1 cell wall.precursor synthesis** |  |  |  |
| UGE1 (UDP-D-glucose/ UDP-D-galactose 4-epimerase 1) | 1,1 | 1.39E-02 | At1g12780 |
| **10.6 cell wall.degradation** |  |  |  |
| BXL2 (Beta-xylosidase 2) | 1,49 | 6.81E-03 | At1g02640 |
| BXL1 (Beta-xylosidase 1) | 1,48 | 2.58E-02 | At5g49360 |
| Polygalacturonase, putative | -1,28 | 8.46E-03 | At1g60590 |
| **10.7 cell wall.modification** |  |  |  |
| XTR3 (Xyloglucan endo­transglycosylase 3) | 1,43 | 6.42E-03 | At5g57550 |
| ATEXPB1 (Arabidopsis thaliana expansin B1) | -1,04 | 7.87E-03 | At2g20750 |
| ATEXPA8 (Arabidopsis thaliana expansin A8) | -1,23 | 1.38E-02 | At2g40610 |
| XTR8 (Xyloglucan endo-trans­glycosylase-related 8) | -1,79 | 1.66E-02 | At3g44990 |
| **11.1 lipid metabolism.fatty acid synthesis and elongation** |  |  |  |
| MCCA (Methylcrotonoyl-CoA carboxylase) | 1,64 | 8.04E-04 | At1g03090 |
| **11.3 lipid metabolism.phospholipid synthesis** |  |  |  |
| Methyltransferase/ Phosphoethanolamin-N-Methyltransferase | -1,5 | 7.41E-03 | At1g73600/02 |
| **11.9 lipid metabolism.lipid degradation** |  |  |  |
| Glycerophosphoryl diester phosphor­diesterase family-Protein | 1,71 | 6.42E-03 | At5g41080 |
| **12.3 N-metabolism.N-degradation** |  |  |  |
| GDH2 (Glutamate Dehydrogenase 2) | 1,33 | 2.74E-03 | At5g07440 |
| **13.1 amino acid metabolism.synthesis** |  |  |  |
| ASN1 (Glutamine-dependent asparagine synthetase 1) | 2,18 | 6.42E-03 | At3g47340 |
| Small subunit of Acetolactate-Synthase, putative | 1,14 | 6.81E-03 | At2g31810 |
| **13.2 amino acid metabolism.degradation** |  |  |  |
| ERD5 (Early responsive to dehydration 5); Proline-Dehydrogenase | 2,15 | 2.74E-03 | At3g30775 |
| **14 S-assimilation** |  |  |  |
| APR3 (APS reductase 3); Adenylyl-Sulfate-Reductase | -1,34 | 1.34E-02 | At4g21990 |
| APR1 (APS reductase 1); Adenylyl-Sulfate-Reductase | -1,68 | 6.42E-03 | At4g04610 |
| **15.2 metal handling.binding, chelation and storage** |  |  |  |
| NAS1 (Nicotineamine synthase 1) | -1,09 | 1.34E-02 | At5g04950 |
| **16.8 secondary metabolism.flavonoids** |  |  |  |
| CYP706A5 | -1,19 | 2.19E-02 | At4g12310/20 |
| **17.2 hormone metabolism.auxin** |  |  |  |
| DRM1_DYL1 (Dormancy-associated protein-like 1) | 1,71 | 7.79E-03 | At1g28330 |
| Dormancy/ auxin associated family-Protein | 1,41 | 1.44E-02 | At2g33830 |
| Auxin-responsive-Protein, putative | -1,06 | 4.64E-02 | At2g21210 |
| **17.5 hormone metabolism.ethylene** |  |  |  |
| ACS6 (1-aminocyclopropane-1-carboxylic acid (ACC) synthase 6) | -1,03 | 6.42E-03 | At4g11280 |
| **20.1 stress.biotic** |  |  |  |
| Disease resistance-Protein (TIR-NBS class), putative | -1,14 | 2.16E-02 | At1g72910/30 |
| RPP4 (recognition of peronospora parasitica 4) | -1,19 | 2.74E-03 | At4g16860 |
| **20.2 stress.abiotic** |  |  |  |
| DNA heat shock-Protein, putative | 1,39 | 1.64E-02 | At2g17880 |
| Similar to Dehydration-responsive protein | -1,11 | 6.42E-03 | At3g56080 |
| **26 misc** |  |  |  |
| BGAL1 (Beta galactosidase 1) | 1,27 | 1.43E-02 | At3g13750 |
| CYP89A2 | -1,01 | 3.94E-02 | At1g64900 |
| UDP-glucoronosyl/ UDP-glucosyl transferase family-Protein | -1,04 | 2.56E-02 | At1g06000 |
| ATACP5_PAP17; acid Phosphatase/ Serin/Threonin-Phosphatase | -1,07 | 9.82E-03 | At3g17790 |
| ATGH9C2 (Arabidopsis thaliana glycosyl hydrolase 9C2) | -1,07 | 2.09E-02 | At1g64390 |
| CYP96A4 | -1,08 | 2.66E-02 | At5g52320 |
| Short-chain dehydrogenase/ reductase (SDR) family-Protein | -1,18 | 1.09E-02 | At3g61220 |
| FMO GS-OX1 (Flavin-monooxygenase glucosinolate S-oxygenase 1) | -1,43 | 3.30E-02 | At1g65860 |
| CYP79F2 | -1,49 | 4.19E-02 | At1g16400/10 |
| FMO GS-OX3 (Flavin-monooxygenase glucosinolate S-oxygenase 3) | -1,51 | 2.01E-02 | At1g62560 |
| **27.3 RNA.regulation of transcription** |  |  |  |
| Ovule development-Protein, putative | 1,61 | 1.25E-02 | At1g79700 |
| CCA1 (Circadian Clock Associated 1) | -1,05 | 1.11E-02 | At2g46830 |
| TOC1 (Timing of CAB expression 1-like-Protein), putative | -1,19 | 2.66E-02 | At2g46670/790 |
| Zinc finger (B-box type) family-Protein | -1,58 | 1.63E-02 | At2g21320 |
| **29.4 protein.posttranslational modification** |  |  |  |
| Serin/Threonin-Proteinkinase, putative | 1,8 | 6.42E-03 | At3g59350 |
| β-2-subunit of 5'-AMP-activated-Proteinkinase, putative | 1,26 | 7.41E-03 | At5g21170 |
| Protein kinase family-Protein | 1,15 | 6.42E-03 | At4g38470 |
| ATSR1 (Arabidopsis thaliana serine/threonine protein kinase 1) | -1,04 | 1.31E-02 | At5g01820 |
| **29.5 protein.degradation** |  |  |  |
| Zinc finger (C3HC4-type RING finger) family-Protein | 1,36 | 2.80E-02 | At5g22920 |
| SCPL34; serine-type-Carboxypeptidase | 1,04 | 7.41E-03 | At5g23210 |
| BT5 (BTB and TAZ domain protein 5) | 1 | 8.14E-03 | At4g37610 |
| Subtilase family-Protein | -1,05 | 7.79E-03 | At5g59130 |
| **30.5 signalling.G-proteins** |  |  |  |
| AtRABG3B_RAB7_RAB75_RABG3B, RAS-related protein | 1,29 | 6.81E-03 | At1g22740 |
| **31.1 cell.organisation** |  |  |  |
| Male sterility MS5 family-Protein | -1,55 | 1.25E-02 | At1g04770 |
| **33 development** |  |  |  |
| SEN1 (Senescence 1) | 2,5 | 6.42E-03 | At4g35770 |
| COL2 (constans-like 2); transcription factor, binds zinc ions | -1,18 | 1.09E-02 | At3g02380 |
| Nodulin MtN3 family-Protein | -1,27 | 1.68E-03 | At5g50800 |
| **34.2 transport.sugar** |  |  |  |
| STP1 (Sugar transporter 1) | 1,67 | 6.51E-03 | At1g11260 |
| **34.4 transport.nitrate** |  |  |  |
| NTP3 (Nitrate transporter 3) | -1,28 | 1.18E-02 | At3g21670 |
| **34.9 transport.metabolite transporter at the mitochondrial membrane** |  |  |  |
| Glycerin-3-Phosphate-Transporter, putative | -1,13 | 4.77E-02 | At3g47420 |
| **35 not assigned** |  |  |  |
| Unknown protein | 1,5 | 4.16E-02 | At1g33055 |
| Unknown protein | 1,42 | 3.30E-02 | At4g27450 |
| Unknown protein | 1,39 | 1.31E-02 | At3g15630 |
| BTB/POZ domain-containing-Protein | 1,24 | 8.14E-03 | At2g30600 |
| Unknown protein | 1,22 | 1.29E-02 | At1g54740 |
| ACT domain-containing-Protein | 1,21 | 6.20E-03 | At2g39570 |
| Unknown protein | 1,18 | 3.50E-03 | At2g42040 |
| BTB/POZ domain-containing-Protein | 1,17 | 6.42E-03 | At2g30600 |
| Unknown protein | 1,08 | 2.16E-02 | At1g22890 |
| PAR1 (PHY rapidly regulated 1) | 1,06 | 2.15E-02 | At2g42870 |
| Glycosyl hydrolase family 17-Protein | 1,04 | 6.51E-03 | At4g18340 |
| Unknown protein | 1,01 | 3.06E-02 | At1g11380 |
| Unknown protein | -1 | 1.66E-02 | At5g67370 |
| NHL12 | -1,1 | 1.31E-02 | At2g35960 |
| Unknown protein | -1,11 | 9.79E-03 | At5g55620 |
| Unknown protein | -1,2 | 1.33E-02 | At1g13650 |
| Unknown protein | -1,32 | 1.95E-02 | At5g01790 |
| LSU2 (Response to low sulfur 2) | -1,44 | 7.79E-03 | At5g24660 |
