# Supplementary material for: A Naturally Associated Rhizobacterium of Arabidopsis thaliana Induces a Starvation-Like Transcriptional Response while Promoting Growth
Source: PLoS One. 2011 Dec 28;6(12):e29382. doi: 10.1371/journal.pone.0029382 (PMC3247267; doi:10.1371/journal.pone.0029382)
Supplement: Table S3 — BBCH scale for evaluation of plant development. (DOCX) [file pone.0029382.s006.docx]

Supporting Information Table S3:

BBCH scale for Arabidopsis

**Number Stage**

1. 1. leaf unfolded
2. 2. leaf unfolded
3. 3. leaf unfolded
4. 4. leaf unfolded
5. 5. leaf unfolded
6. 6. leaf unfolded
7. 7. leaf unfolded
8. 8. leaf unfolded
9. 9. leaf and more unfolded
10. flower buds present, enclosed by leaves
11. flower buds visible from above
12. 1. internode of main inflorescence visible
13. flowers raised above youngest leaves
14. 1. petals visible, flowers still closed
15. 1. open flower
16. 2 to 5 open flowers
17. 1. flower with stile longer than petals
18. 2 to 5 flowers with stile longer than petals
19. 1. pod has reached final size
20. 2 to 5 pods have reached final size
21. > 5 pods have reached final size
22. style of 1. flower of axillary inflorescence longer than petals
23. 1. Pod of secondary inflorescence reached final size
