# Supplementary material for: A Naturally Associated Rhizobacterium of Arabidopsis thaliana Induces a Starvation-Like Transcriptional Response while Promoting Growth
Source: PLoS One. 2011 Dec 28;6(12):e29382. doi: 10.1371/journal.pone.0029382 (PMC3247267; doi:10.1371/journal.pone.0029382)
Supplement: Table S6 — List of genes that are down-regulated by P. sp. G62, but upregulated by sugar starvation. (DOCX) [file pone.0029382.s009.docx]

Supporting Information Table S6: List of genes that are down-regulated by *P. sp*. G62, but upregulated by 3 h of sugar starvation (Osuna D, Usadel B, Morcuende R, Gibon Y, Blasing OE, et al. (2007) Temporal responses of transcripts, enzyme activities and metabolites after adding sucrose to carbon-deprived Arabidopsis seedlings. Plant Journal 49: 463-491.)

|  | **AGI** | **6h P. sp. G62** | **sugar starvation** | **Annotation** |
| --- | --- | --- | --- | --- |
| 258181_at | At3g21670 | -1,283942196 | 2,006219476 | Major facilitator superfamily protein |
| 264931_at | At1g60590 | -1,279155112 | 2,036824796 | Pectin lyase-like superfamily protein |
| 254835_s_at | At4g12310 At4g12320 | -1,194082977 | 1,271038071 | CYP706A5__cytochrome P450, family 706, subfamily A, polypeptide 5 |
| 245448_at | At4g16860 | -1,188169858 | 1,69201861 | RPP4__Disease resistance protein (TIR-NBS-LRR class) family |
| 258497_at | At3g02380 | -1,183465591 | 1,769651499 | ATCOL2_COL2__CONSTANS-like 2 |
| 262374_s_at | At1g72910 At1g72930 | -1,142696924 | 1,400803917 | Toll-Interleukin-Resistance (TIR) domain-containing protein |
| 266719_at | At2g46830 | -1,049577043 | 1,140416937 | CCA1__circadian clock associated 1 |
| 262882_at | At1g64900 | -1,013355875 | 1,882261984 | CYP89_CYP89A2__cytochrome P450, family 89, subfamily A, polypeptide 2 |
| 246998_at | At5g67370 | -1,003820652 | 1,298715912 | Protein of unknown function (DUF1230) |
| 263252_at | At2g31380 | -0,977506335 | 1,402029178 | STH__salt tolerance homologue |
| 245450_at | At4g16880 | -0,890428186 | 1,081578154 | Leucine-rich repeat (LRR) family protein |
| 259275_at | At3g01060 | -0,832612886 | 1,154615793 | unknown protein |
| 253835_at | At4g27820 | -0,818455159 | 1,505664301 | BGLU9__beta glucosidase 9 |
| 249774_at | At5g24150 | -0,811174001 | 2,095652006 | SQE5_SQP1__FAD/NAD(P)-binding oxidoreductase family protein |
| 256603_at | At3g28270 | -0,798274629 | 3,023796772 | Protein of unknown function (DUF677) |
| 260380_at | At1g73870 | -0,727945918 | 1,644431398 | B-box type zinc finger protein with CCT domain |
| 255088_at | At4g09350 | -0,723659681 | 1,319185973 | Chaperone DnaJ-domain superfamily protein |
| 255016_at | At4g10120 | -0,696329588 | 1,972636092 | ATSPS4F__Sucrose-phosphate synthase family protein |
| 245734_at | At1g73480 | -0,629808602 | 1,274972474 | alpha/beta-Hydrolases superfamily protein |
| 251727_at | At3g56290 | -0,626564375 | 1,52690578 | unknown protein |
